# Everybody's Page

**Published:** 1889-10-12

**Authors:** 


### Everybody's Page.

About 3,125,000lb. of wool are exported every year from South and South-Western Persia.

Formerly gum tragacanth came from Constantinople alone, but now the Red Sea ports are entering into competition, and a great deal is shipped from Bagdad, Beyrout, and Mersine. All the produce of Syria and Arabia goes to these ports.

California is developing a wine trade, and though its wines stand second to those of France the demand for them is steadily increasing. Most Californian wine is shipped to Europe by way of Cape Horn, and if well fermented is improved by the voyage.

One of the specifics of the Chinese pharmacopeia consists of the following pleasing ingredients: dried snakes, pulverised, 1oz.; wasps and their nests, ½oz.; centipedes, 3oz.; scorpions, 6oz.; and toads, 10oz. These are ground to powder, mixed with honey, and made into pills.

As children will insist on eating all that they should not, it sometimes happens that in country districts we hear of poisoning by laburnum. The symptoms appear within half an hour after the seeds have been swallowed, but may be checked by administering an emetic of sulphate of zinc.

The ruby, the sapphire, the topaz, the emerald, and the amethyst, are practically the same chemically. They are generically known as corundum, and consist of oxide of aluminium, called alumina, in an almost pure state, with just traces of metallic oxides, which give the different tints to the varieties of crystals.

Yet another hypnotic has been given to the world by Professor Neering, of Strasbourg. It is called chloralamide, and when given in a dose of 60 grains or less produces, within half-an-hour, sleep that lasts from seven to nine hours. It is said to have no material effect on the circulation. Its price is about three times as much as that of chloral.

Though the name of Daniel Lambert has become a synonym for obesity, very few people know how much he weighed. When he died in 1809, at the age of forty, his weight was 52st. 11lb. The coffin, with his body in it, could not be brought down the stairs of the house in which he died, and the wall at the sides of the window had to be broken away to permit of its being taken out.

In France, if a patient who is under chloroform shows any signs of heart failure, those in attendance hold him head downwards till he is restored. The method is said never to fail; and so convinced are some surgeons of its efficacy, that they have operating-tables made in such a fashion that one end can be elevated at a moment's notice, and the patient be practically made to stand on his head for an instant or two.

The idea of treating dipsomania as a disease is not a new one. It was stated by Wirtzung, in a book published in 1617. He puts drunkenness next to the plague in the catalogue of diseases, and recommends a treatment similar to that in vogue at the present day. In earlier days, however, this view did not prevail. An early Dutch method with female inebriates was to suspend them by a pulley and duck them three times in the nearest horsepond. Charlemagne's method was of a still more heroic kind. For the first offence the drunkard was tortured privately. A second was punished by public torture. If this did not effect a cure, and a third relapse occurred, the offender was killed. This is one way of effecting a decrease of drunkenness.

Russian engineers are busy establishing the electric light in Central Asia.

Formerly when a whale was killed on the Orcadian coast, the natives were in the habit of pickling the whale-beef for food, and those who have tasted it say that it was better than might have been supposed.

More than half the sugar used in the world is made from beetroot. The manufacture began to be important little more than twenty years ago. Germany began it, and France, Austria, Russia, and the United States have followed the Teutons' example.

The American Indians are said to be very successful in their treatment of small-pox. They keep their patients in dark rooms, and allow a current of air to pass continually over his body. They also administer a decoction of some herb, but the nature of this they keep secret.

The botanical name of Ylang-Ylang is Unona Odoratissima, while the meaning of its native appellation is "flower of flowers." It is the blossom of a large tree indigenous to the Indian Archipelago. Its colour is a greenish-yellow, and the odour, which resembles a combination of jasmine and lilac, is so powerful that it scents the air for miles around.

From ten to twelve ounces a day is the amount of meat required for a healthy adult who takes an ordinary amount of work and exercise. As a rule women eat less than this, and delude themselves with the idea that they don't require as much food as men, even when they work as hard. Hence it is that so many women drift into invalidism in middle age.

A new industry is being carried on in Brazil, the distilling of paraffin from peat. About ten beds are being worked at Marslin, and though the digging has now reached a depth of 150ft., there is no sign of the supply being exhausted. About 300 hands are employed, and 33 boilers are in use. The paraffin thus obtained is used in the manufacture of candles. We commend this idea to Irish philanthropists.

While the question of smoking is being discussed in the newspapers, it may be worth while to quote a law which was passed in New Jersey in 1883: "Hereafter no person or persons in this State shall knowingly sell any cigarette or cigarettes, or tobacco in any of its forms, to any minor under the age of sixteen years." This would at least tend to prevent that youthful smoking which is physically the most injurious.

One of the most curious results of the introspective habits of our age is the recently-published journal of Marie Bashkirtseff, a young Russian artist, who died in 1884 at the age of twenty-five. For thirteen years she had kept a diary, in which every incident and emotion are ruthlessly set down. She does not spare her own weaknesses and vanities; she is self-conscious to the last degree. She tells how she went to see Bastien-Lepage painting in Gambetta's death-chamber, and how the scene made her weep. Most women would have ended there. Not so Marie Bashkirtseff. She goes on: "But he had turned his back, intent on his painting. So I, not liking to lose the benefit of my sensibility, hastily stretched my hand towards him, and went out, my face covered with tears. I hope he noticed it." The diary is, as the writer herself said, interesting as a human document, but as the record of a woman's life it is sad and humiliating. A woman who never forgets herself is a creature painful to contemplate.

